# Global review and guidelines to avoid opportunistic predation of birds and bats in mist nets

**DOI:** 10.1002/ece3.10390

**Published:** 2023-07-31

**Authors:** Guilherme Wince de Moura, Karen Mustin, Fernando Antonio Silva Pinto, Sylvia Coelho Alves Sineiro, Bruna da Silva Xavier, Luciana Moraes Costa, Carlos Eduardo Lustosa Esbérard, Alexeia Barufatti, William Douglas Carvalho

**Affiliations:** ^1^ Programa de Pós‐Graduação em Biodiversidade e Meio Ambiente Universidade Federal da Grande Dourados (UFGD) Dourados Brazil; ^2^ Programa de Pós‐Graduação em Biodiversidade Tropical Universidade Federal do Amapá (UNIFAP) Macapá Brazil; ^3^ Department of Biodiversity, Ecology and Evolution Complutense University of Madrid Madrid Spain; ^4^ Instituto Nacional da Mata Atlântica (INMA) Santa Teresa Brazil; ^5^ Programa de Pós‐Graduação Educação em Ciências e Saúde Universidade Federal do Rio de Janeiro Rio de Janeiro Brazil; ^6^ Programa de Pós‐Graduação em Ecologia Universidade Federal do Rio de Janeiro (UFRJ) Rio de Janeiro Brazil; ^7^ Laboratório de Ecologia de Mamíferos, Departamento de Ecologia, Instituto de Biologia Universidade do Estado do Rio de Janeiro Rio de Janeiro Brazil; ^8^ Laboratório de Diversidade de Morcegos, Departamento de Biologia Animal, Instituto de Biologia Universidade Federal Rural do Rio de Janeiro Seropédica Brazil; ^9^ Terrestrial Ecology Group (TEG‐UAM), Departamento de Ecología, Facultad de Ciencias Universidad Autónoma de Madrid Madrid Spain; ^10^ Centro de Investigación en Biodiversidad y Cambio Global (CIBC‐UAM) Universidad Autónoma de Madrid Madrid Spain

**Keywords:** avian, Chiroptera, mist‐netting protocols, opportunistic behavior, Passeriforme, safe sampling, trophic interaction

## Abstract

Mist nets are one of the most widely used techniques in the study of birds and bats worldwide. However, a number of risks are involved, including opportunistic predation. Given this potential cost, here we: (1) review the global literature to understand the factors that might contribute to predation risk for birds and bats captured in mist nets; (2) review existing guidelines for best practice use of mist nets; and (3) based on our reviews, recommend new guidelines for the use of mist nets to minimize the risk of opportunistic predation. Based on keyword in English, Spanish, Portuguese, and French, and using Google Scholar, Scopus, SciElo, and Web of Science, we found 48 articles reporting opportunistic predation. In the included articles, 178 predation events, involving 52 predator and 84 prey species, were reported. In most of the reports, the mist nets were placed at ground level, the bats and birds were preyed on from the shelf closest to the ground, the mist‐net checks occurred at intervals of 1 h or 30 min and the most common predators were arboreal and scansorial species (primates and marsupials). Despite the occurrences of predation in 13 countries, guidelines for best practice mist‐net use were found in only three, despite extensive searches and contact with key people in each country. Based on the existing guidelines and our results, we recommend that mist nets be fixed with the lowest shelf at least 50 cm above ground level and be checked at 15‐min intervals; when predators are observed near mist nets, the nets either be constantly observed, closed, or relocated; suppressed the vegetation around the mist nets; captured animals be removed from the mist nets as soon as possible, and more than one researcher/technician should be in the field at all times.

## Graphical abstract

Mist nets are one of the most widely used techniques in the study of birds and bats worldwide. However, a number of risks are involved, including opportunistic predation. Using guidelines for the correct use of mist nets can not only decrease predation rates but also the risk of injury to birds and bats.

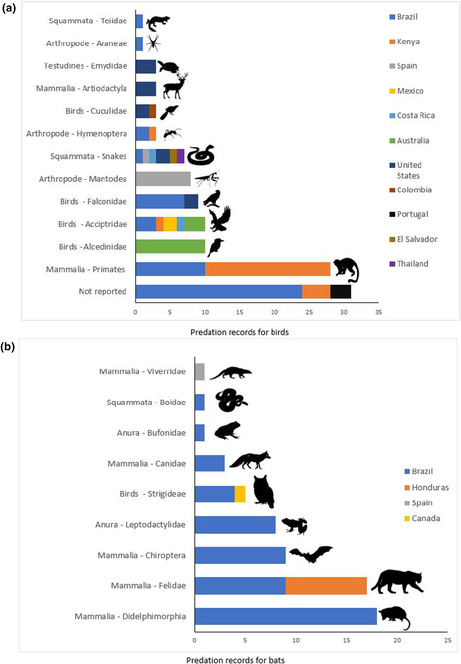

## 1 INTRODUCTION

The capture and handling of wild animals are necessary for studies in diverse areas of science, for example, where morphometric data need to be collected or in studies which aim to monitor populations through tagging or the use of radio collars (Wilson & McMahon, 2006). For birds and bats, the capture of individuals using mist nets is one of the most widely and frequently used methods in studies of their ecology, populations, and life histories (e.g., Beja et al., 2010; Carvalho et al., 2018; Freitas et al., 2020; Keyes & Grue, 1982; Soares et al., 2020). Mist‐net use is so widespread in part because they represent a low‐cost, easy‐to‐implement, and efficient method of capture (Kunz & Kurta, 1988; Spotswood et al., 2012).

Despite being highly efficient, mist nets do introduce taxonomic bias. For example, for Neotropical bats, this method predominantly captures Phyllostomidae, which are more frequently caught as they have less accurate echolocation than other families (e.g., Vespertilionidae, Molossidae, and Emballonuridae) and therefore do not detect the mesh of the mist nets (Carvalho et al., 2023; Kunz & Kurta, 1988; Kunz & Parsons, 2009; Sampaio et al., 2003). For birds, mist nets are the most efficient capture method, though their use is largely limited to population studies and biometry that do not aim to quantify bird diversity, where fixed point count surveys, and line transects are more effective (Costa‐Braga et al., 2014; Munn, 1991; Whitman et al., 1997). Although mist nets are effective in sampling some groups of birds and bats, this method is considered invasive and can cause injuries such as strangulation, fractures in tarsals and metatarsals, and skin cuts and can result in fatalities either due to these injuries or via opportunistic predation (Breviglieri & Pedro, 2010; Serra‐Gonçalves et al., 2017; Spotswood et al., 2012).

Despite being underreported, opportunistic predation is recognized as one of the major causes of death in birds caught in mist nets (see Clewley et al., 2018; Spotswood et al., 2012) and is also expected to be so for bats (see Serra‐Gonçalves et al., 2017). Opportunistic predation is when a potential predator takes advantage of the fact that its prey is unable to react (in this case, entangled in the mesh of the mist net), attacking it, killing it, and consuming it whole or in parts (e.g., Breviglieri & Pedro, 2010; Carvalho et al., 2016; Ruiz‐Esparza et al., 2012; Serra‐Gonçalves et al., 2017; Spotswood et al., 2012). Different opportunistic predators of birds and bats have been reported in the literature, among them ants, spiders, amphibians, reptiles (including lizards and snakes), birds, opossum, wild and domestic canids and cats, primates, and bats (Breviglieri & Pedro, 2010; Brito et al., 2007; Brooks, 2000; Carvalho et al., 2011, 2016; Castro et al., 2011; Clewley et al., 2018; Curcino et al., 2009; Gallego et al., 2021; Gazarini et al., 2008; Hilário et al., 2017; Legal et al., 2018; Melo et al., 2018; Novaes et al., 2010; Ross, 2010; Serra‐Gonçalves et al., 2017).

Despite underreporting of opportunistic predation in mist nets, several studies have recommended ways to reduce the risk, such as cutting the vegetation around the mist nets, reducing the intervals between mist‐net checks to disentangle the animals, and raising the lowest shelf of the mist nets to confirm that the net will not touch the ground (see Carvalho et al., 2016, 2018; Gallego et al., 2021; Hilário et al., 2017; Serra‐Gonçalves et al., 2017). In addition, countries such as Brazil, Australia, England, and the United States recommend, through the government or associations, that mist nets should be not only monitored at regular intervals of at least 30 min but, if possible, also continuously monitored so that animals can be removed immediately after capture (e.g., Barlow, 1999). However, many megadiverse countries where mist nets are used, such as Kenya and Colombia, seemingly have no standardized guidelines for researchers and students working with birds and bats.

Given the importance of mist nets as a scientific method for studying birds and bats, the quantification and characterization of opportunistic predation records reported in the literature are extremely important as they will allow for the formulation of global guidelines of best practice for the use of mist nets, which in turn could contribute to reducing this type of injury. Thus, our objectives here were to (1) globally qualify and quantify opportunistic predation events in birds and bats captured in mist nets, based on a global review of studies that reported this type of predation; (2) characterize existing guidelines on the use of mist nets from countries in which predation records were reported; and (3) based on our findings, recommend new best practice guidelines for the sampling of birds and bats with mist nets to reduce the risks of opportunistic predation.

## 2 METHODS

This work is based on a global review of opportunistic predation, in addition to data on opportunistic predation of bats from sampling carried out by Laboratório de Diversidade de Morcegos (i.e., “*Bat Diversity lab*”) (LADIM) of the Universidade Federal Rural do Rio de Janeiro (UFRRJ) between 1989 and 2013.

### 2.1 Systematic literature review

**FIGURE 1:**
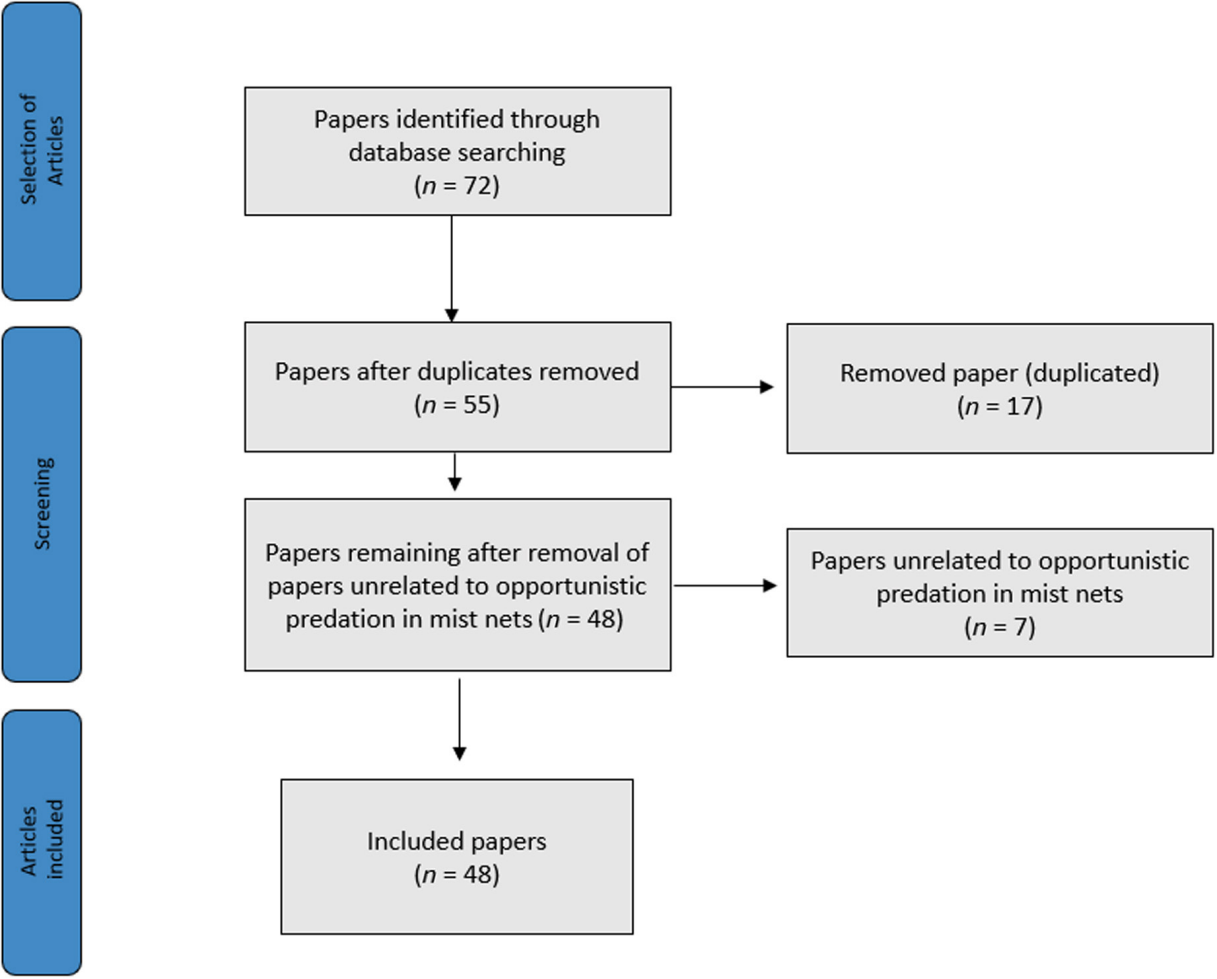
Flowchart showing how the screening of articles on opportunistic predation in mist nets was carried out.

We systematically compiled and reviewed studies of opportunistic predation of birds and bats caught in mist nets. To do so, we searched for studies that were published using the following bibliographic databases: Google Scholar (https://scholar.google.com/), Scopus (https://www.scopus.com/search/form.uri?display=basic#basic), Scientific Electronic Library Online (SciELO—https://scielo.org/en) and Web of Science (https://clarivate.com/webofsciencegroup/solutions/web‐of‐science/), using the keywords: (“Predation” OR “Opportunistic predation” OR “Mortality”) AND (“mist net” OR “mist‐netting”) AND (“Chiroptera” OR “Bat*” OR “Bird” OR “Passeriformes”). The search was carried out with the keywords in Portuguese, English, French, and Spanish, as together these languages represent four of the five most widely spoken languages in the world in terms of the number of countries where they are spoken. The other language of the five most spoken is Arabic, which it was not possible to include in this study, but which we believe did not bias the result. Subsequently, the studies found were examined and filtered, removing duplicates and those that were not related to opportunistic predation of birds and bats in mist nets. The number of studies excluded and retained after each of the screening steps was retained according to the Preferred Reported Items for Systematic Reviews and Meta‐Analyses statement (Moher et al., 2009; Figure 1).

After screening, the following data were extracted from the complied studies: (a) country where the predation occurred, (b) height (relative to the ground) where the predation occurred, (c) height of the first shelf (the one closest to the ground) of the mist net relative to the ground, (d) length of time (hereafter referred to as interval) between mist‐net checks, (e) predator and (f) prey species, and (g) whether any pre‐established guidelines were used to define the methodology implemented. We subsequently searched for existing guidelines and protocols regarding the use of mist nets in each of the countries where records of opportunistic predation were reported using these same bibliographic databases.

### 2.2 Data on opportunistic predation of bats captured by LADIM

Given that opportunistic predation of bats in mist nets seems to be underreported, we also obtained unpublished data on these events from LADIM. We compiled the records of opportunistic predation in sampling nights between 1989 and 2013 in the Brazilian states of Bahia, Espírito Santo, Goiás, Mato Grosso, Minas Gerais, Pará, Rio de Janeiro, Roraima, and São Paulo. These samplings were carried out with mist nets (dimensions: 6 × 3 m, 9 × 3 m, 12 × 3 m; 14 mm mesh) that were set up from ground level (the first shelf of the mist net, or lowest shelf, was located just above the ground) on trails, roads and at the exit of day shelters (hollow trees, caves, roofs, and abandoned buildings). The mist nets remained open from before sunset (~15–20 min) until midnight or until dawn of the following day. The intervals between mist‐net checks were of approximately 20 min and the LADIM samplings did not follow any pre‐established guidelines.

## 3 RESULTS

### 3.1 Status of knowledge on the opportunistic predation of birds and bats in mist nets

**FIGURE 2:**
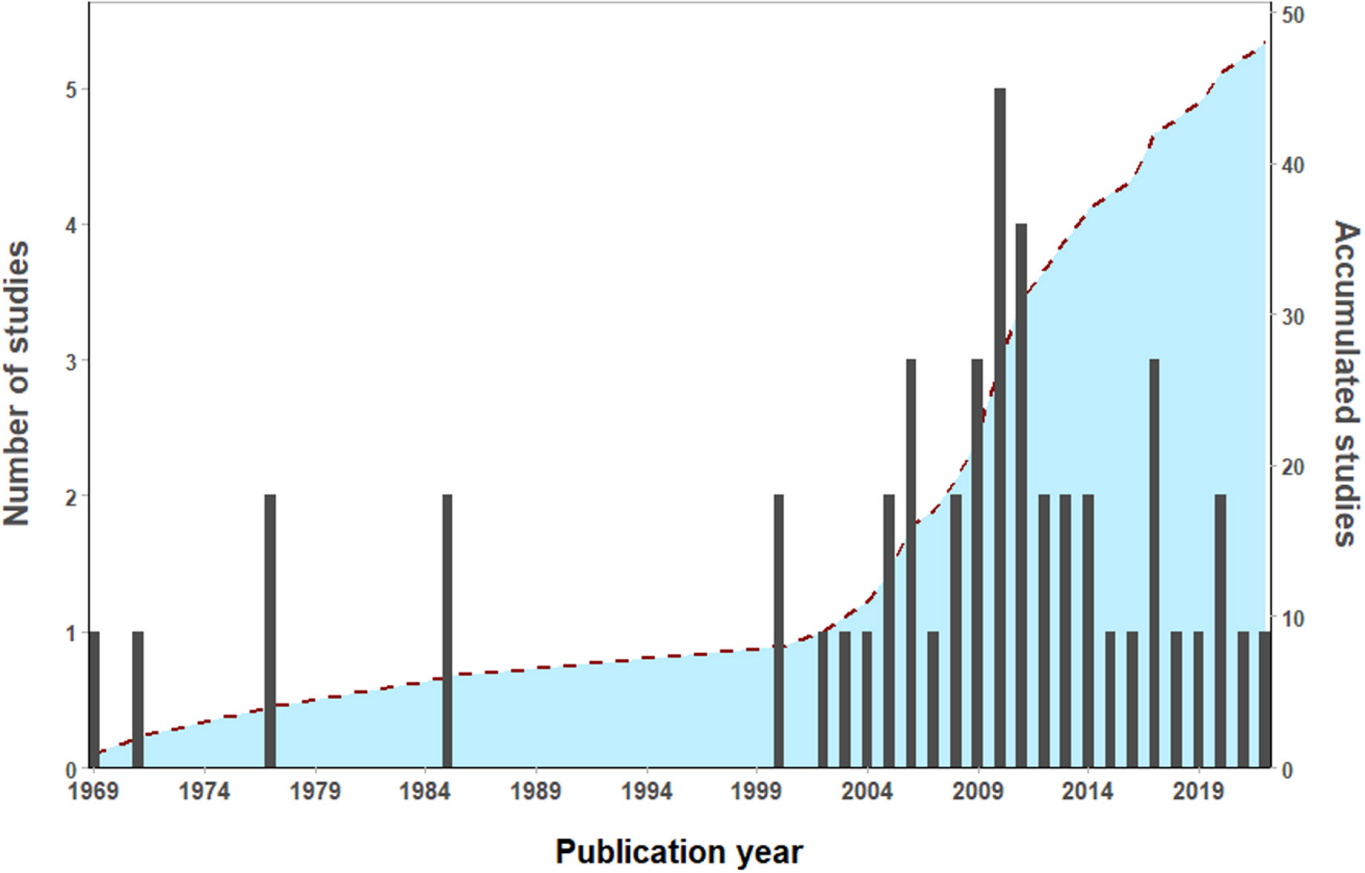
Annual number of studies that recorded opportunistic predation by birds and bats in mist nets. The shaded area shows the cumulative number of studies.

**FIGURE 3:**
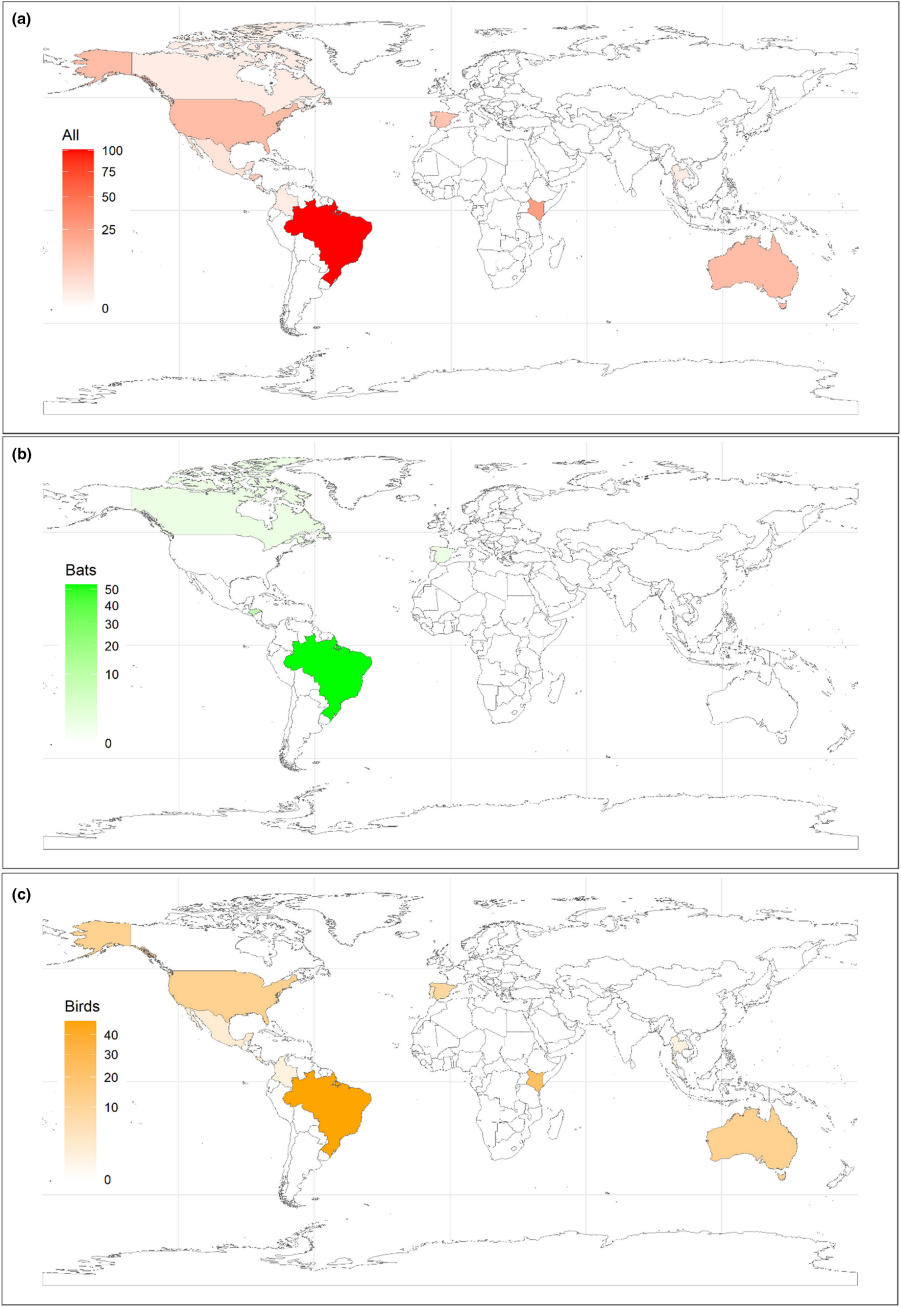
Opportunistic predation records found in different countries: (a) Total opportunistic predation records; (b) opportunistic predation records on bats; and (c) opportunistic predation records on birds.

Our searches returned a total of 72 studies between 1969 and 2022. Of this total, 17 were excluded for being duplicates and 7 for being studies unrelated to opportunistic predation in mist nets (Figure 1). Thus, 48 studies were retained for the analyses, which collectively reported 178 different predation events involving 52 species of predator and 84 species of prey (Tables S1 and S2). Opportunistic predation records were reported between the years 1969 and 2022 in 13 countries: Australia, Brazil, Canada, Colombia, Costa Rica, El Salvador, Spain, United States, Honduras, Mexico, Portugal, Kenya, and Thailand (Figures 2 and 3). Of the 48 studies, 26 (54.0%) were related to opportunistic predation of birds and 22 (46.0%) of bats (Table S1). One hundred and fifteen (64.6%) of the 178 opportunistic predation events reported involved birds, and 63 (35.4%) involved bats.

### 3.2 Methodological considerations in mist‐net use related to opportunistic predation

Of the 178 opportunistic predation records, 39 (21.9%) reported the mist‐net shelf in which the event occurred (17 for birds and 22 for bats). For birds, 12 events (70.6%) occurred in the first shelf (closest to the ground), two (11.8%) in the second shelf, and three (17.6%) in the third shelf. For bats, 17 (77.3%) occurred in the first shelf, four (18.2%) in the second shelf, and one (4.5%) in the third shelf.

Eighteen of the 178 records (10.1%) reported the height at which the mist net was set in relation to the ground (4 for birds and 14 for bats). For birds, in one case (25%) the mist net was positioned at ground level and in three cases (75%) at 30 cm from the ground. For bats, in eight cases (57.1%) the mist net was positioned at ground level, in one case (7.1%) at 40 cm from the ground, and in five cases (35.7%) at 50 cm from the ground.

Ninety‐six records (73 for birds and 23 for bats) were accompanied by information on the interval between mist‐net checks. For birds, 42 events (57.5%) occurred when mist nets were checked at one‐hour intervals, 28 (38.4%) at 30‐min intervals, and three (4.1%) at 20‐min intervals. For bats, one record (4.4%) occurred in a study where the mist nets were checked only once every 2 h, in five cases (21.7%) nets were checked every hour, 13 (56.5%) every 30 min, and four (17.4%) every 15 min.

### 3.3 Prey and predators

Of the 178 opportunistic predation records, 154 specified the prey species (98 for birds and 56 for bats). These included 63 bird species and 21 bat species. Most of the predated birds belong to the Order Passeriformes (*n* = 89). Bird species' of the orders Columbiformes (*n* = 4), Piciformes (*n* = 3), Coraciiformes (*n* = 1), and Galliformes (*n* = 1) were also found to be predated. Most of the predated bats were Phyllostomidae (45 records). Bat species' of the orders Molossidae (*n* = 4), Vespertilionidae (*n* = 4), and Miniopteridae (*n* = 1) were also found to be targets of opportunistic predation.

**FIGURE 4:**
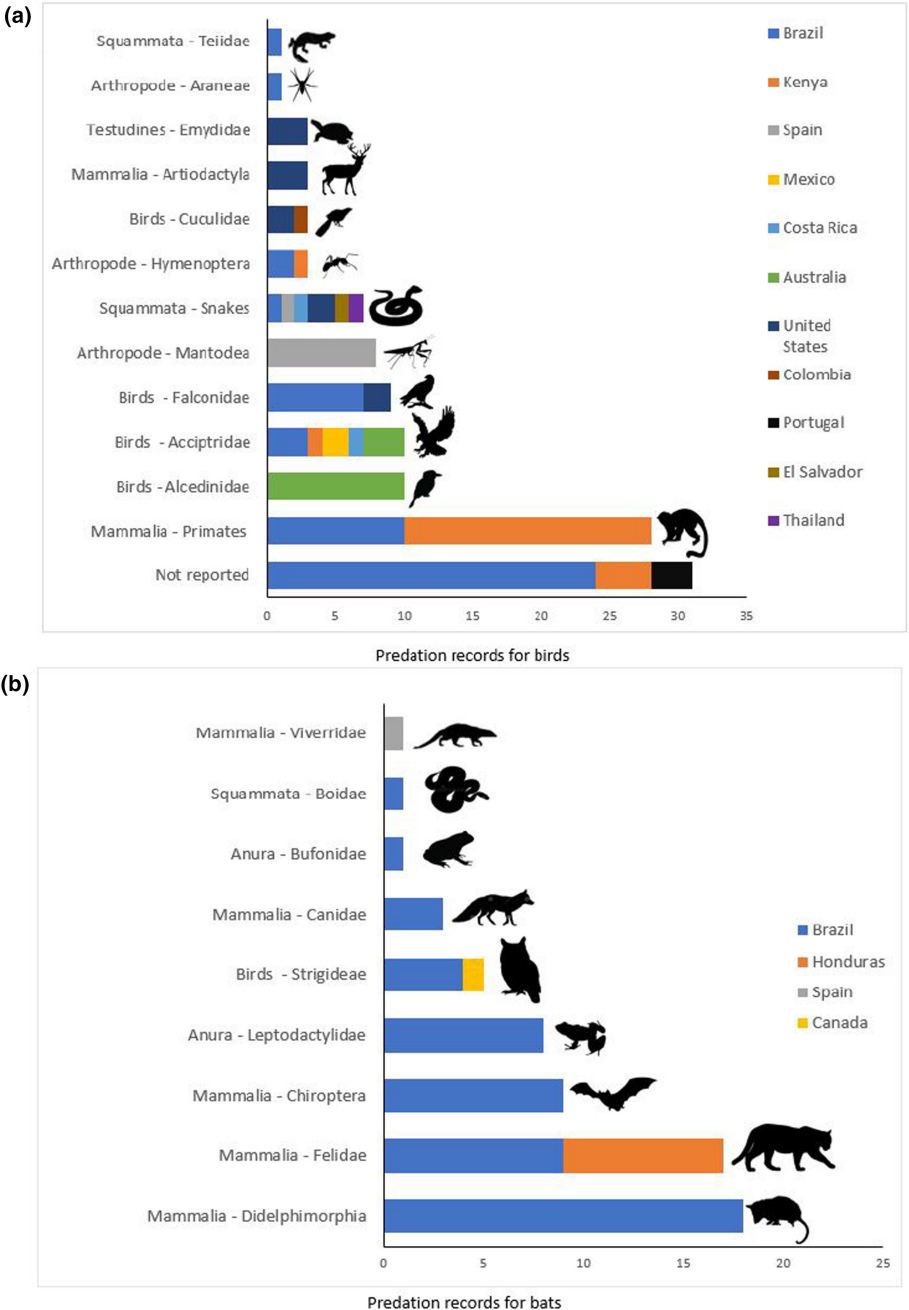
Total records of opportunistic predation of birds (a) and bats (b) in relation to different taxonomic categories of predators in different countries.

Of the 178 opportunistic predation records, 147 specified the predator species involved (84 for birds and 63 for bats). These included 29 species predating birds, mainly primates, passerine birds and birds of prey, with some records of predation by herpetofauna and invertebrates (Figure 4; Table S2), and 23 species predating bats, most of which were mammals, and particularly marsupials, felines, and bats, as well as some records of predation by owls and herpetofauna (Figure 4; Table S2).

### 3.4 Unpublished data on opportunistic predation of bats

Between April 1989 and May 2013, we found 38 records of opportunistic predation from bat sampling carried out by LADIM, which represented 0.1% of all captured animals (41,492 captures). Also, these records represent 9.5% of all bats that suffered some type of injury in these samples (*n* = 401 injured and/or dead bats). The species *Carollia perspicillata* (*n* = 12) was the most preyed bat species, followed by *Artibeus lituratus* (*n* = 7), *Molossus molossus* (*n* = 7), *Sturnira lilium* (2), and *Desmodus rotundus* (*n* = 2). The other nine species had only one predation record each. The spectacled owl *Pulsatrix perspicillata* (*n* = 12), the marsupial *Didelphis aurita* (*n* = 5), and the domestic cat *Felis catus* (*n* = 4) were the predators with the most records, followed by the marsupial *Monodelphis* sp. (*n* = 1) and the bat *Phyllostomus hastatus* (*n* = 4). It was not possible to identify the species of predator of the other bat species, as the predation was noted when the researchers found the predated animal in the mist nets. LADIM only recorded the height at which predation occurred in the mist net for seven predated bats: four predation events occurred in the second shelf and three events in the third shelf, with all bats having been predated by the spectacled owl *P. perspicillata*.

### 3.5 Mist‐netting guidelines in countries with records of opportunistic predation

Guidelines were found for the following countries where there were records of opportunistic predation: Brazil (*Manual de Anilhamento de Aves Silvestres*— De Sousa & Serafini, 2020); United States (Handbook of Field Methods for Monitoring Landbirds—Ralph et al., 1993, and The North American Banders Study Guide—The North American Banding Council, 2001) and Australia (Australian Bird Bander's Manual—Lowe, 1989). No guidelines were found for Colombia, Costa Rica, El Salvador, Spain, Mexico, Portugal, or Kenya.

The Wild Bird Banding Manual (*Manual de Anilhamento de Aves Silvestres* in Portuguese) indicates that banders are responsible for the safety and well‐being of the studied birds and summarizes considerations to minimize risks, including (1) being efficient in terms of the time spent handling individuals, to reduce the potential for harm to the individual itself as well as the time other individuals are waiting to be processed; (2) not capturing or handling animals in adverse weather conditions such as heavy rain and/or excessive heat; (3) that traps or nets be closed when predators are seen close to the area and/or when the risk of predation is higher; (4) that mist nets be “frequently” checked, but without specifying what constitutes being frequent; and (5) to never leave mist nets open without any supervision (Manual de Anilhamento de Aves Silvestres—De Sousa & Serafini, 2020).

The Guidelines in the Handbook of Field Methods for Monitoring Landbirds (USA), while not describing anything about opportunistic predation, suggests that mist nets should be checked every 45 min, with this time not exceeding 1 h (Ralph et al., 1993). In addition, they recommend that the rounds should be more frequent when the weather is less favorable, such as on very hot days (Ralph et al., 1993). The North American Banders Study Guide recommends placing the mist nets so that the shelf closest to the ground is off the ground, otherwise, birds trapped in this shelf can become entangled in vegetation, drown in puddles that can form due to flooding and rain, get soaked by dew or be attacked by insects (mainly ants), or even struggle against the ground and suffer some type of injury (The North American Banding Council, 2001). This guideline also emphasizes the fact that birds and other animals that are entangled in the mist nets attract predators, which can cause injury and death to the animals and damage the nets (The North American Banding Council, 2001). In addition, another factor mentioned concerns the frequency of mist‐net checks, emphasizing that once the nets are installed, they must be checked frequently, every 20 or 30 min, increasing this frequency when the weather is hot, cold, wet, and windy (The North American Banding Council, 2001).

The Australian Bird Bander's Manual presents a series of guidelines for action when the bander perceives that there is a risk of opportunistic predation, such as checking the mist nets more frequently, closing or moving them to other locations, and fixing them in a higher position (Lowe, 1989). These guidelines also highlight the main opportunistic predators of birds in the region, such as Kookaburras (*Dacelo novaeguineae*), corvids, and currawongs, the latter of which, as soon as they find the mist nets, keep watching and attack any bird that is tangled in the mesh (Lowe, 1989). Thus, this guideline indicates the closure or displacement of mist nets when there is an occurrence of other animals near the mist nets if it is not possible to maintain constant observation of these potential opportunistic predators (Lowe, 1989). This guideline also describes that other birds that may occasionally attack birds entangled in mist nets are birds of prey, with these being discouraged by the experience of also becoming entangled, not returning to attempt further predation (Lowe, 1989). In addition to giving examples of invertebrate predators, Lowe (1989) also mentions ant attacks on any entangled bird species when the mist nets are in contact with the ground. The recommendation to avoid this type of ant predation is to keep the mist nets high enough so that the entangled birds are above the ground (Lowe, 1989). Lowe (1989) also indicates that opportunistic predations can also be caused by reptiles and domestic animals, mainly dogs.

## 4 DISCUSSION

Our results show that the opportunistic predation of birds and bats in mist nets is widespread across countries on different continents. However, some countries (e.g., Brazil and Kenya) had more opportunistic predation records than others (e.g., Colombia and Thailand), mainly for birds. This result indicates that in Brazil, for example, researchers report more opportunistic predations that occur in mist nets (e.g., Breviglieri & Pedro, 2010; Carvalho et al., 2016; Curcino et al., 2009; Gallego et al., 2021; Guimarães et al., 2020; Serra‐Gonçalves et al., 2017). Also, the results compiled from long‐term sampling carried out by LADIM reinforce that predation of this kind is more common than reporting levels would suggest, and by extension that globally, there seems to be a large gap for this type of record.

We found that opportunistic predation occurs in 0.1% of bats captured by LADIM with mist nets, being also responsible for 9.5% of the causes of injuries and deaths of bats. The LADIM data reveal that in just one laboratory working in one country, there are 38 unpublished occurrences of opportunistic bat predation over a 24‐year period, which represents more than half of the number of published records of opportunistic bat predations found by our review worldwide (63 records). In their review, Serra‐Gonçalves et al. (2017) had already described the low number of publications of this type in relation to the high capture rate for bats in tropical forests. Data from the USA and Canada have shown mortality rates of 0.23% for birds caught in mist nets, with opportunistic predation as one of the main causes (Spotswood et al., 2012). While opportunistic predation was also the main cause cited, data from the UK suggest much lower mortality rates of 0.001, with juveniles particularly susceptible and an increase in predation risk in the winter (Clewley et al., 2018). Therefore, our global review shows only a small portion of the opportunistic predations that truly must occur in studies using mist nets, and it is important that future reporting of predation events include the total number of animals captured so that robust quantitative estimates of the impact of opportunistic predation can be made. Although there is no acceptable rate for injured or dead animals that are captured with mist nets (Spotswood et al., 2012), the correct and safe use of invasive methods for capturing wild animals is of fundamental importance (Ralph et al., 1993; Sikes & the Animal Care and Use Committee of the American Society of Mammalogists, 2016). Thus, we conclude that this type of predation can and should be reduced by using guidelines that learn from problems and errors that have occurred in the past. In addition, reporting this type of antagonistic event becomes very important for the development of new guidelines and the improvement of existing ones, which will improve the safety and efficiency of the capture method (Serra‐Gonçalves et al., 2017).

Considering the data for birds and bats together, as they showed similar patterns, we found that opportunistic predation was proportionally higher in the first shelf of the mist nets. In addition, most of the authors who reported the height at which the mist nets were fixed, described that the first shelves (closest to the ground) were close to the ground. Bats are most captured in mist nets between 0.7 and 2.4 m high, with the highest capture rate in shelves 2 and 3, with shelf 1 (closest to the ground) showing the lowest capture rate (see Carvalho et al., 2018). Considering that terrestrial mammals were the main opportunistic predators of birds (e.g., *Cercopithecus mitis*) and bats (e.g., *Leopardus wiedii*), by increasing the height at which the mist nets are fixed, the predation rate will tend to decrease (Carvalho et al., 2018; Serra‐Gonçalves et al., 2017). For example, Carvalho et al. (2018) recommend that mist nets can be fixed at least 50 cm from the ground. Also, the guidelines of The North American Banding Council (2001) and Lowe (1989) describe that mist nets should be high enough so that predation by ants does not occur. However, the height of the net from the ground may be lower according to the objective of each study (see Carvalho et al., 2018). Also, to check whether the height of the first shelf is sufficient to avoid any predation event, the researcher or technician in the field can throw an ~150 g object (e.g., cloth bag) into this shelf and check if the object touches the ground (see Carvalho et al., 2016). If the object touches the ground, the height of the shelf must be increased (Carvalho et al., 2016).

The height at which the mist nets are set up, together with the interval between mist‐net checks, has already been shown by different studies to be the main driver of the occurrence of opportunistic predation in birds and bats (see Carvalho et al., 2016; Gallego et al., 2021; Guimarães et al., 2020; Serra‐Gonçalves et al., 2017). We found a marked variation in time between mist‐net checks. Although different studies and guides recommend the interval between 15 and 45 min for mist‐net checks (e.g., Carvalho et al., 2016; Ralph et al., 1993; Serra‐Gonçalves et al., 2017), our results showed that approximately 50% of the studies that reported this time, left the mist nets alone for more than 45 min. Depending on the type of predator seen in the vicinity before starting sampling, mist‐net check of a maximum of 15 min should be recommended (see Carvalho et al., 2016; Serra‐Gonçalves et al., 2017). However, when a decrease in the interval between mist‐net checks is not sufficient to prevent opportunistic predation, more restrictive actions can be considered. For example, seven of the 12 predation records made by the spectacled owl *Pulsatrix perspicillata* in a rainforest area in Brazil (LADIM data) were recorded in a single sampling night. Thus, in the case of viewing predators such as owls or currawongs (Lowe, 1989), we recommend that researchers remain close to the mist nets. Alternatively, researchers can move mist nets or cancel sampling nights as predators learn the location of the nets and which animals are most vulnerable to being entangled (see Gallego et al., 2021).

The prey most reported by the studies we found are species considered common and abundant, having a wide geographic distribution and, consequently, end up being the most captured species in different regions (e.g., *Eurillas latirostris* for Kenya and *Turdus leucomelas* and *Carollia perspicillata* for Brazil—Giraudo et al., 2008; Tchoumbou et al., 2020; Costa et al., 2021). Birds were predated mainly by primates, passerine birds, and birds of prey, while marsupials and wild and domestic cats being the main predators of bats. Predators such as *Cercopithecus mitis* and *Pulsatrix perspicillata* have a generalist diet and have a wide distribution and/or high local abundance (Coleman & Hill, 2015; Orihuela‐Torres et al., 2018). In general, the main opportunistic predators of birds (primates) and bats (marsupials) are classified as arboreal and scansorial species, respectively (Wilman et al., 2014). Thus, in addition to the fact that these species have generalist habits and high local abundance, they can take advantage of branches and vegetation that are in contact with or close to the mist nets to capture birds or bats entangled in the nets (Breviglieri & Pedro, 2010; Ruiz‐Esparza et al., 2012). To prevent these animals from accessing the mist nets, the vegetation around the mist nets must be cut (e.g., Hilário et al., 2017), reducing the chance of these potential predators accessing birds and bats that are entangled in the mist nets.

Among the nine countries where we found records of opportunistic predation, only the United States (The North American Banding Council, 2001; Ralph et al., 1993) and Australia (Lowe, 1989) have guidelines that indicate how to prevent animals from being preyed on mist nets. We also found that none of the studies reviewed have indicated that they used any of these guidelines for setting up the mist nets, disentangling and handling the captured animals. In fact, all the guidelines evaluated by us provide information on how to minimize and avoid accidents when trapping animals with mist nets, such as keeping the first shelf of the mist net off the ground and shortening the interval between mist‐net checks (see Lowe, 1989; The North American Banding Council, 2001; Ralph et al., 1993). However, these guidelines are directed toward birds, with bats having no specific guidelines. Thus, we consider that if most studies had followed the recommendations of these guidelines, as well as other studies (e.g., Carvalho et al., 2016; Gallego et al., 2021; Guimarães et al., 2020; Serra‐Gonçalves et al., 2017), many predation records could have been avoided. Therefore, the opportunistic predation rate we found, even for the LADIM data, could be lower if the different studies had used previous recommendations. However, for different recommendations to be more effective and change according to the purpose of the study, thus minimizing injuries to birds and bats caused by predation, it is necessary for researchers to start reporting this type of predation as a matter of routine. When describing these predation events, researchers should also provide, where possible, the following information: height and shelf of the mist nets where predation occurred, estimated time of predation, species of prey and predator, the time between mist‐net checks, number of people in the field, and actions taken after the predation event. It would be useful to create systemized, international databases for this type of reporting.

We found that opportunistic predation of birds and bats in mist nets is not geographically biased. However, our results show that the number of records published worldwide does not represent the real number of occurrences of opportunistic predation in mist nets. In addition, this type of predation may be occurring due to the nonuse of different recommendations and guidelines that can be found in the literature. (e.g., Carvalho et al., 2016; Gallego et al., 2021; Guimarães et al., 2020; Lowe, 1989; The North American Banding Council, 2001; Ralph et al., 1993; Serra‐Gonçalves et al., 2017). While it is not possible to eliminate the risk of opportunistic predation, we have an ethical responsibility to reduce this risk as much as possible. For this, it is important to follow the best practices for assembling and handling mist nets. Therefore, based on our results, we recommend the following guidelines to be followed when sampling birds and bats with mist nets:

- Fix the mist nets at least 50 cm above the ground, checking that the first shelf (closest to the ground) does not touch the ground when throwing an object weighing approximately 150 g into the net (Carvalho et al., 2016).
- Check mist nets at intervals not exceeding 15 min (Carvalho et al., 2016; Serra‐Gonçalves et al., 2017).
- If a potential predator is sighted near the mist net, further decrease the interval between mist‐net checks. When necessary, keep a researcher close to the mist nets to scare away potential predators. In extreme cases, change the sampling location or cancel sampling for that day/night (Gallego et al., 2021).
- Suppress the vegetation around the mist net to reduce the access that arborial and scansorial species have to the nets (Hilário et al., 2017).
- Remove the animals entangled in the mist nets as soon as possible to avoid the vocalization of these animals, which can draw the attention of potential predators (The North American Banding Council, 2001). In addition, rapid removal of the animals will reduce stress on them and injury risk, as well as reducing damage to the mist nets, especially in the case of bats.
- Avoid carrying out field activities with the use of mist nets with only one researcher/technician. With a greater number of people in the field, there will be no work overload in places with many captures of birds and bats. Thus, birds and bats will be disentangled more quickly, not being vulnerable to opportunistic predation for long periods of time.

Finally, the use of these guidelines for the correct use of mist nets can not only decrease predation rates but also the risk of injury to birds and bats, thus making the method safer. However, these guidelines can be improved with the increase in publications of this type of record. In other words, researchers who work with mist nets should publish their records of opportunistic predation (e.g., as a short communication or natural history note, even if in local/regional journals), providing all possible information, including the total number of animals captured so that quantitative estimates of mortality can be calculated and ultimately so that other researchers can improve the use of this sampling method. We also strongly recommend that these guidelines (and new ones that may be created) should be widely discussed, based on the reality of each country/region, disseminated, and published, mainly by associations and societies that aim at research with birds and bats.

## AUTHOR CONTRIBUTIONS

**Guilherme Wince de Moura:** Conceptualization (equal); data curation (equal); formal analysis (equal); investigation (equal); methodology (equal); writing – original draft (equal); writing – review and editing (equal). **Karen Mustin:** Conceptualization (equal); data curation (equal); formal analysis (equal); funding acquisition (equal); investigation (equal); methodology (equal); project administration (equal); supervision (equal); validation (equal); writing – original draft (equal); writing – review and editing (equal). **Fernando Antonio Silva Pinto:** Conceptualization (supporting); methodology (supporting); visualization (supporting); writing – review and editing (supporting). **Sylvia Coelho Alves Sineiro:** Conceptualization (supporting); investigation (equal); methodology (equal); writing – review and editing (supporting). **Bruna da Silva Xavier:** Methodology (supporting); writing – review and editing (supporting). **Luciana Moraes Costa:** Conceptualization (supporting); methodology (supporting); writing – review and editing (supporting). **Carlos Eduardo Lustosa Esbérard:** Conceptualization (equal); data curation (equal); funding acquisition (equal); investigation (equal); methodology (equal); writing – review and editing (equal). **Alexeia Barufatti:** Conceptualization (equal); investigation (equal); methodology (equal); visualization (equal); writing – original draft (equal); writing – review and editing (equal). **William Douglas Carvalho:** Conceptualization (equal); data curation (equal); formal analysis (equal); funding acquisition (equal); investigation (equal); methodology (equal); project administration (equal); supervision (equal); writing – original draft (equal); writing – review and editing (equal).

## FUNDING INFORMATION

Coordenação de Aperfeiçoamento de Pessoal de Nível Superior (CAPES—Financing Code 001); Conselho Nacional de Desenvolvimento Científico e Tecnológico (CNPq—301061/2007‐6); Spanish Ministry of Universities (CA3/RSUE/2021‐00197).

## CONFLICT OF INTEREST STATEMENT

No potential conflict of interest is reported by the authors.

## Supporting information


Table S1

## Data Availability

All relevant data are within the manuscript and its Supporting Information files.
