# Supplementary material for: Global review and guidelines to avoid opportunistic predation of birds and bats in mist nets
Source: Ecol Evol. 2023 Jul 31;13(8):e10390. doi: 10.1002/ece3.10390 (PMC10388402; doi:10.1002/ece3.10390)
Supplement: Supplementary file 1 — Table S1 [file ECE3-13-e10390-s001.docx]

(Supplementary Material – Table 1) - List of 48 articles analyzed with opportunistic predation in mist nets.

| **Paper title** | **Autor** | **Kind** |
| --- | --- | --- |
| Anuros preying bats | Esbérard et al., 2005 | Bat |
| New Evidence of Bat Predation by the Woolly False vampire bat *Chrotopterus auritus* | Nogueira et al., 2006 | Bat |
| Bat predation by *Phyllostomus hastatus* | Oprea et al., 2006 | Bat |
| Snakes preying on bats: new records from Brazil and a review of recorded cases in the Neotropical Region | Esbérard & Vrclbraci, 2007 | Bat |
| Predações oportunísticas de morcegos por *Didelphis albiventris* no sul do Brasil | Gazarini et al., 2008 | Bat |
| Ataques oportunísticos da cuíca (*Philander frenatus*) a morcegos em redes de neblina | Patrício-Costa et al., 2008 | Bat |
| Opportunistic predatory behavior of margay, *Leopardus wiedii* (Schinz, 1821), in Brazil | Rocha-Mendes & Bianconi, 2009 | Bat |
| Predação oportunista de morcegos por *Cerdocyon thous* (Carnivora, Canidae) no sudeste do Brasil | Novaes et al., 2010 | Bat |
| Predação de *Tadarida brasiliensis* por *Chrotopterus auritus* no sul do Brasil | Brito et al., 2010 | Bat |
| Predação de morcegos (Phyllostomidae) pela cuíca d’água *Chironectes minimus* (Zimmermann, 1780) (Didelphimorphia, Didelphidae) e uma breve revisão de predação em Chiroptera | Breviglieri & Pedro, 2010 | Bat |
| Predação oportunista de *Artibeus planirostris* (Spix, 1823) e *Carollia perspicillata* (Linnaeus, 1758) (Chiroptera, Phillostomidae) por marsupiais e anuro na APA do Rio Curiaú, Amapá, Brasil | Castro et al., 2010 | Bat |
| Predação oportunística de *Molossus molossus* (Pallas, 1766) (Chiroptera: Molossidae) por  *Rhinella jimi* (Stevaux, 2002)(Anura: Bufonidae) na Caatinga, Pernambuco, Brasil | Silva et al., 2010 | Bat |
| Predation on Broad-eared bat *Nyctinomops laticaudatus* by the Spectacled Owl *Pulsatrix perspicillata* in southwestern Brazil | Carvalho et al., 2011 | Bat |
| Opportunistic Consumption of Blood from pallas's long-tongued bat, *Glossophaga soricina*, by the Common Vampire, *Desmodus rotundus*, in Brazil | Souza et al., 2011 | Bat |
| Opportunistic predation of a little brown rat (*Myotis lucifugus*) by a great horned owl (*Bubo virginianus*) in southern Yukon | Jung et al., 2011 | Bat |
| Opportunistic predation on bats trapped in mist nets by *Leptodactylus vastus* (Anura: Leptodactylidae) | Filho et al., 2013 | Bat |
| Opportunistic predation by Crested owl *Lophostrix cristata* upon Seba’s Short-tailed  Bat *Carollia perspicillata* | Rocha & Lopez-Baucells, 2014 | Bat |
| Opportunistic Predation of *Artibeus lituratus* (Chiroptera, Phyllostomidae) by the Marsupial *Marmosops incanus* (Didelphidae, Didelphimorphia) in the Atlantic Forest, Minas Gerais | Faria & Barros, 2014 | Bat |
| Predation on bats by genets (Genetta genetta) Linneaus, 1758): a review | Mas et al., 2015 | Bat |
| Opportunistic predation of a silky short-tailed bat (*Carollia brevicauda*) by a tawny-bellied screech-owl (*Megascops watsonii*), with a compilation of predation events upon bats entangled in mist-nets | Serra-Gonçalves et al., 2017 | Bat |
| Opportunistic predation events of bats entangled in mist nets by margay *Leopardus wiedii*  (Schinz, 1821) in northwest Honduras: recommendations to avoid preventable casualties | Gallego et al., 2021 | Bat |
| Opportunistic predation of *Carollia brevicauda* (Schinz, 1821) (Chiroptera: Phylostomidae) by *Marmosa demerarae* (Thomas, 1905) (Marsupialia: Didelphidae) in the Brazilian Amazon | Alencastre-Santos et al., 2022 | Bat |
| Copperhead kills netted birds. | Hubbard, 1969 | Bird |
| Predation on a netted bird by Smooth-billed Anis | Gill & Stokes 1971 | Bird |
| Sparrow Hawk predation on Bank Swallows | Freer, 1973 | Bird |
| Further Evidence of White-tailed Deer Eating Birds in Mist Nets | Allan, 1978 | Bird |
| Roadrunner takes birds from mist net | Barclay, 1977 | Bird |
| Recher notes. | Recher, 1985 | Bird |
| Predation on Birds Caught in Mist-Nets in Upland Kenyan Forest Fragments | Brooks, 2000 | Bird |
| Causes and Frequency of Deaths among Birds Mist-Netted for Banding Studies at Two Localities. | Armstrong, 2000 | Bird |
| Bird ringing on Mira region (Beira Litoral, Portugal). | Petronilho, 2002 | Bird |
| Predation on Birds by the white hawk (*Leucopternis albicollis*) | Komar, 2003 | Bird |
| Notes on a collection of amphibians and reptiles from El Salvador. | Leenders & Colwell, 2004 | Bird |
| *Terrapene carolina carolina* (Eastern Box Turtle) Predation | Swarth, 2005 | Bird |
| Praying Mantis killing passerines in mist nets | Bigas et al., 2006 | Bird |
| Predação oportunística de passeriforme em rede-de-neblina por indivíduo de *Rupornis magnirostris* (Falconiformes: Accipitridae) | Curcino et al., 2009 | Bird |
| Reticulated Python predation on a silver pheasant in Khao Vainational park, Thailand | Sukumal & Savini, 2009 | Bird |
| *Oxybelis fulgidus*: Diet. | Van Dort, 2011 | Bird |
| Predation of Birds trapped in Mist Nets by Raptors ins the Brazilian Caatinga | Ruiz-Esparza, 2012 | Bird |
| *Tupinambis merianae* (Squamata: Sauria: Teiidae): tentativa de predação de *Turdus leucomelas* em redes de neblina | Santos & Silva, 2012 | Bird |
| Captura accidental de *Malpolon monspessulanus* en una red japonesay primera cita de *Sylvia atricapilla* en la dieta de la especie | Villarán et al., 2013 | Bird |
| Opportunistic predation of a Common Scale-backed Antbird (*Willisornis poecilinotus*) by a Goliath bird-eating spider (*Theraphosa blondi*) in the Eastern Brazilian Amazon | Carvalho et al., 2016 | Bird |
| Praying mantis eating the feathers of a passerine caught in a mist-net | Illa & Jutglà, 2017 | Bird |
| Predation of birds in mist nets by callitrichids (primates): how to prevent similar events | Hilário et al., 2017 | Bird |
| Opportunistic predation on birds trapped in mist nets in two areas in the Atlantic Forest of southeastern Brazil | Melo et al., 2018 | Bird |
| Registro da predação de ave em rede de neblina por Leucopternis kuhli (Aves: Accipitridae)  na Floresta Nacional do Tapajós | Anjos et al., 2019 | Bird |
| Birds trapped in mist nets killed by opportunistic predators in a forest ins Southwestern Amazonia | Guimarães et al., 2020 | Bird |
| First record of a Semiplumbeous hawk (*Leucopternis semiplumbeus*) preying on a Redthroated ant-tanager (*Habia fuscicauda*) in Tirimbina biological reserve, Costa Rica | Villegas, 2020 | Bird |

**References**

Allan, T. A., 1978. Further Evidence of White-tailed Deer Eating Birds in Mist Nets. Bird Banding, 49: 184.

Alencastre-Santos, Correia, L. L., Souza, L. M., Silva, C. R., Vieira, T. B., 2022. Opportunistic predation of *Carollia brevicauda* (Schinz, 1821) (Chiroptera: Phylostomidae) by *Marmosa demerarae* (Thomas, 1905) (Marsupialia: Didelphidae) in the Brazilian Amazon. Mammalia, 86: 347-350. doi: 10.1515/mammalia-2021-0083

Anjos, G. H., Castro, R. A. B., Lopes, E. V., 2019. Registro da predação de ave em rede de neblina por *Leucopternis kuhli* (Aves: Accipitridae) na Floresta Nacional do Tapajós. Atualidades Ornitológicas, 209: 27

Armstrong, T., 1985. Frequence of Deaths among Birds Mist-Netted for Banding Studies at Two Localities. Wildlife Research, 12(2): 321-326.

Barclay, J.S. 1977. Roadrunner takes bird from mist net. Bird-Banding 48:280.

Bigas, D., Piccardo, J. & Copete, J. L. 2006. Praying Mantis killing passerines in mistnets. Dutch Birding 28: 237–238.

Breviglieri, C.P.B., Pedro, W.A., 2010. Predação de morcegos (Phyllostomidae) pela cuíca d’água *Chironectes minimus* (Zimmermann, 1780) (Didelphimorphia, Didelphidae) e uma breve revisão de predação em Chiroptera. Chiroptera Neotropical 16: 732–739.

Brito, J.E.C., Gazarini, I.P., Bernardi, C., 2007. Predação oportunística de morcegos por *Didelphis albiventris,* (Lund 1840) Chiroptera Neotropical. 14(2): 408-411.

Brooks, T., 2000. Predation on birds caught in mist nets in upland Kenyan forest fragments. Wilson Bull. 112(2): 292-294. [https://doi.org/10.1676/0043-5643(2000)112[0292:POBCIM]2.0.CO;2](https://doi.org/10.1676/0043-5643(2000)112%5b0292:POBCIM%5d2.0.CO;2).

Carvalho, L.F.A.C., Cunha, N.L., Santos, C.F., 2011. Predation on Broad-eared bat *Nyctinomops laticaudatus* by the Spectacled Owl *Pulsatrix perspicillata* in southwestern Brazil. Revista Brasileira de Ornitologia, 19: 417-418.

Carvalho, W. D., Norris, D. Michalski, F., 2016. Opportunistic predation of a Common Scale-backed Antbird (*Willisornis poecilinotus*) by a Goliath bird-eating spider (*Theraphosa blondi*) in the Eastern Brazilian Amazon, Studies On Neotropical Fauna and Environment. 51(3): 239-241. <https://doi.org/10.1080/01650521.2016.1237802>

Castro, I.J., Silva, C.R.., Costa, A.J.S., Martins, A.C.M., 2011. Predação oportunista de *Artibeus planirostris* (Spix, 1823) e *Carollia perspicillata* (Linnaeus, 1758) (Chiroptera, Phyllostomidae) por marsupiais e anuro na APA do Rio Curiaú, Amapá, Brasil. Acta Amazonica. 41(1): 171-174. <https://doi.org/10.1590/S0044-59672011000100020>.

Curcino, A., Heming, N.M., Feraboli, A., 2009. Predação oportunística de passeriformes em rede-de-neblina por indivíduo de *Rupornis magnirostris* (Falconiformes: Accipitridae). Atualidades Ornitológicas, 151: 22.

Esbérard, C. E. L., Jordão, T., Costa, L. M., Nergallo, H. G., 2005. Anuros Preying bats.

Esbérard, C. E. L., & Vrcibradic, D., 2007. Snakes preying on bats: new records from Brazil and a review of recorded cases in the Neotropical Region. Revista Brasileira de Zoologia, 24(3): 848-853. <https://doi.org/10.1590/S0101-81752007000300036>

Faria, M. B. 2014. Opportunistic Predation of *Artibeus lituratus* (Chiroptera, Phyllostomidae) by the Marsupial *Marmosops incanus* (Didelphidae, Didelphimorphia) in the Atlantic Forest, Minas Gerais. Chiroptera Neotropical. 20: 1297-1300.

Filho, L. E., Feijó, A., Rocha, P. A., 2014. Opportunistic predation on bats trapped in mist nets by *Leptodactylus vastus* (Anura: Leptodactylidae). Biotemas, 27(3): 205-208. <https://doi.org/10.5007/2175-7925.2014v27n3p205>

Freer, V. M., 1973. Sparrow Hawk Predation on Bank Swallows. The Wilson Bulletin, 85(2): 231–33.

Gallego, Z.L., Van Berkum, P.M., Martin, T., 2021. Opportunistic predation events of bats entangled in mist nets by margay *Leopardus wiedii* (Schinz, 1821) in northwest Honduras: recommendations to avoid preventable casualties. Journal of Bat Res. & Conservation 14(1):,33-36. <http://dx>.doi.org/10.14709/BarbJ.14.1.2021.04.

Gazarini, J., Brito, J.E.C., Bernardi, I.P., 2008. Predações oportunísticas de morcegos por *Didelphis albiventris* no sul do Brasil. Chiroptera Neotropical. 14, 408- 411.

Gill, B., & Stokes, C. C., 1971. Predation on a netted bird by Smooth-hilled Anis. The Wilson Bulletin, 83(1): 101

Guimarães, D. P., Lima, J., Souza, V. L., Guilherme, E., 2020. Birds trapped in mist nets killed by opportunistic predators in a forest in Southwestern Amazonia. Revista Brasileira de Zoociências 21(1): 1-8. <https://doi.org/10.34019/2596-3325.2020.v21.28868>.

Hilário, R.R., Silva, C., Santos, Jr.L.S., Rocha, P.A., Mendes, R.B., Ruiz-Esparza, J., Ferrari, S.F., 2017. Predation of birds in mist nets by callitrichid (primates): how to prevent similar events. Studies on Neotropical Fauna and Environ. 52(2): 168-172. <https://doi.org/10.1080/01650521.2017.1298888>.

Hubbard, J. P. (1969). Copperhead kills netted birds. Eastern Bird Banding Association News 32: 283.

Illa, M. & Roger Jutglàà 2017. Praying mantis eating the feathers of a passerine caught in a mist-net. Revista Catalana d’Ornitologia 33: 50-52.

Jung, T.S., Lausen, C.L., Talerico, J.M., & Slough, B.G., 2011. Opportunistic predation of a little brown bat (*Myotis lucifugus*) by a great horned owl (*Bubo virginianus*) in Southern Yukon. Northwest. Nature*.* 92: 69-72, <https://doi.org/10.1898/10-06.1>.

Komar, O., 2003. Predation on birds by the White Hawk (*Leucopternis albicollis*). Ornitologia Neotropica l14: 541-543.

Leenders T. A. A. M., Watkins-Colwell C. J., 2004. Notes on a collection of amphibians and reptiles from El Salvador. Postilla 231: 1–31

Mas, M., López-baucells., A. & Arrizabalaga, A., 2015. Predation on bats by genets *Genetta genetta*(Linnaeus, 1758): a review. Jounal of Bat Research &Conservation, 8(1): 5-11. <https://doi.org/10.14709/BarbJ.8.1.2015.03>

Melo, M.A., Moreno, D.J., Ribeiro, B.C., Andrade, P.G.B., Magalhães, A.F.A., Carvalho, M.A.S., Piratelli, A.,2018. Opportunistic predation on birds trapped in mist nets in two areas in the Atlantic Forest of southeastern Brazil. Studies On Neotropical Fauna and Environment. 53(2): 1-5. <https://doi.org/10.1080/01650521.2018.1446294>.

Nogueira, M. R., Monteiro, L. R., & Peracchi, A. L., 2006. New evidence of bat predation by the woolly false vampire bat *Chrotopterus auritus*, Chiroptera Neotropical, 12: 286-288.

(1) (PDF) Predators of The Bat Pteropus giganteus Occurring In West Bengal, India. Available from: https://www.researchgate.net/publication/353164939_Predators_of_The_Bat_Pteropus_giganteus_Occurring_In_West_Bengal_India [accessed Jan 31 2023].

Novaes, R. L. M., Menezes, L. F. J., Façanha, A. C. S., Louro, M., Cardoso, T. S., Sant’anna, C., Felix, R. S., Silvares, A., Siqueira, C., Souza, R. F., Oliveira, L. F. C. D., & Aguiar. M. V. P., 2010. Predação oportunista de morcegos por Cerdocyon *thous* (Carnivora, Canidae) no sudeste do Brasil. Chiroptera Neotropical. 16: 29-31.

Oprea, M., Vieira, V. T., Pimenta, P., Mendes, D., Brito, A. D., Ditcheld,L. V., Esbérard, E. L. (2006). Bat predation by *Phyllostomus hastatus*. Chiroptera Neotropical, 12(1): 255-258

Patrício-Costa, P., Pie, M. R., & Passos, F. C. (2010). Ataques oportunísticos da cuíca *Philander frenatus* (Mammalia,Didelphidae) a morcegos em redes de neblina. Chiroptera Neotropical, 16: 41-42

Petronilho, J. M. S., 2002. Bird ringing on Mira region (Beira Litoral, Portugal). Airo 12: 120-124.

Recher, H. F., Gowing, G., & Armstrong, T., 1985. Causes and Frequencey of Deaths among Birds Mist-Netted for Banding Studies at Two Localities. Wildlife Research, 12(2): 321-326.

Rocha-Mendes, F., & Bianconi, G., 2009. Opportunistic predatory behavior of margay, *Leopardus wiedii* (Schinz, 1821), in Brazil. Mammalia. 73: 151–152. <https://doi.org/10.1515/MAMM.2009.017>

Rocha, R., López-Baucells, A., 2014. Opportunistic predation by Crested *Owl Lophostrix cristataupon* Seba’s Short-tailed Bat *Carollia perspicillata*. Revista Brasileira de Ornitologia 22: 35-37. <https://doi.org/10.1007/BF03544230>

Ruiz-Esparza, J., Rocha, P.A., Ribeiro, A., Ferrari, S., 2012. Predation of birds trapped in mist nets by raptors in the Brazilian Caatinga. North American Bird Bander 37(1): 11-17.

Santos, L.S., Vaz-Silva, W., 2012: *Tupinambis merianae* (Squamata: Sauria: Teiidae): tentativa de predação de *Turdus leucomelas* em redes de neblina. Herpetologia Brasileira 1: 35–36.

Serra-Gonçalves, C., López-Baucells, A., & Rocha, R., 2017. Opportunistic predation of a silky short-tailed bat (*Carollia brevicauda*) by a tawny-bellied screech-owl (*Megascops watsonii*), with a compilation of predation events upon bats entangled in mist-nets. Barbastella 10: 1-7, doi:10.14709/BarbJ.10.1.2017.07.

Silva, L. A. M., dos Santos, E. M., & Amorim, F. O. 2010. Predação oportunística de *Molossus molossus* (Pallas, 1766) (Chiroptera: Molossidae) por *Rhinella jimi* (Stevaux, 2002) (Anura: Bufonidae) na Caatinga, Pernambuco, Brasil. Biotemas. 23: 215-218.

<http://10.5007/2175-7925.2010v23n2p215>

Souza, R. F., Sant’Anna, C., Mariana V.P. A., Siqueira, A. C., Tavares, D. C., Laurindo, R. S., & Novaes, R. L. M..2011. Opportunistic consumption of blood from Pallas’s long-tongued bat, *Glossophaga soricina*, by the common vampire, *Desmodus rotundus*, in Brazil. Bat Research News, 52(3): 39–40.

Sukumal, N., & Savini. T 2009. Reticulated python predation on Silver Pheasant in Khao Yai National Park,Thailand. G@llinformed 2: 25–27

Swarth, C. W., 2005. *Terrapene carolina carolina* (Eastern Box Turtle) Predation. Herpetological Review, 36(3): 315

Van Dort, J., 2011. *Oxybelis fulgidus*: Diet. Herpetological Bulletin, 117: 37-38

Villarán, A., Domínguez, J., & Medina, C., 2013. Captura accidental de *Malpolon monspessulanus* en una red japonesa y primera cita de *Sylvia atricapilla* en la dieta de la especie. Boletín de la Asociación Herpetológica Española, 24(2): 18-19.

Villegas, S., 2020. First record of a Semiplumbeous hawk (*Leucopternis semiplumbeus*) preying on a Redthroated ant-tanager (*Habia fuscicauda*) in Tirimbina biological reserve, Costa Rica. El Hornero 35(2): 127-150

(Supplementary Material - Table 2) List of prey/predators recorded in articles selected in the worldwide systematic review on opportunistic predation in mist nets and LADIN’s databases. Not id = Not identified.

| **Predator** | **Prey** | **Kind** | **Reference** |
| --- | --- | --- | --- |
| **Arthropoda** |  |  |  |
| Hymenoptera |  |  |  |
| *Dorylus* | *Pogonocichla stellatai* | Bird | Brooks, 2000 |
| *Eciton burchellii* | *Phlegopsis nigromaculata* | Bird | Guimarães et al., 2020 |
| *Eciton burchellii* | *Dendrocolaptes picumnus* | Bird | Guimarães et al., 2020 |
| Mantodea |  |  |  |
| *Mantis religiosa* | *Erithacus rubecula* | Bird | Bigas et al., 2005;  Marc Illa & Roger Jutglà, 2017 |
| *Mantis religiosa* | *Ficedula hypoleuca* | Bird | Bigas et al., 2005 |
| *Mantis religiosa* | *Cettia cetti* | Bird | Bigas et al., 2005 |
| *Mantis religiosa* | *Phylloscopus trochilus* | Bird | Bigas et al., 2005 |
| *Mantis religiosa* | *Acrocephalus scirpaceus* | Bird | Bigas et al., 2005 |
| Araneae |  |  |  |
| *Theraphosa blondi* | *Willisornis poecilinotus* | Bird | Carvalho et al., 2016 |
| **Anura** |  |  |  |
| Bufonidae |  |  |  |
| *Rhinella jimi* | Not id | Bat | Silva et al., 2010 |
| Leptodactylidae |  |  |  |
| *Leptodactylus labyrinthicus* | *Anoura caudifer* | Bat | Esbérard et al., 2005 |
| *Leptodactylus pentadactylus* | *Carollia perspicillata* | Bat | Castro et al., 2010 |
| *Leptodactylus vastus* | *Glossophaga soricina* | Bat | Filho et al., 2013 |
| *Leptodactylus vastus* | *Tonatia bidens* | Bat | Filho et al., 2013 |
| *Leptodactylus vastus* | *Lonchophylla mordax* | Bat | Filho et al., 2013 |
| *Leptodactylus vastus* | *Myotis nigricans* | Bat | Filho et al., 2013 |
| **Squammata** |  |  |  |
| Boidae |  |  |  |
| *Corallus hortulannus* | *Carollia perspicillata* | Bat | Esbérard & Vrclbraci., 2007 |
| Colubridae |  |  |  |
| *Malpolon monspessulanus* | *Sylvia atricapilla* | Bird | Villarán et al., 2013 |
| *Oxybelis fulgidus* | Humming-bird | Bird | Van Dort, 2011 |
| *Oxybelis fulgidus* | *Dendrocincla homochroa* | Bird | Van Dort, 2011 |
| Pythonidae |  |  |  |
| *Malayopython reticulatus* | *Lophura nycthemera* | Bird | Sukumal & Savini, 2009 |
| Viperidae |  |  |  |
| *Agkistrodon contortrix* | *Thryothorus ludovicianus* | Bird | Hubbard, 1969 |
| *Agkistrodon contortrix* | *Seiurus aurocapillus* | Bird | Hubbard, 1969 |
| Teiidae |  |  |  |
| *Salvator merianae* | *Turdus leucomelas* | Bird | Santos & Silva, 2012 |
| **Testudines** |  |  |  |
| *Terrapene carolina carolina* | *Empidonax virescens* | Bird | Swarth, 2005 |
| *Terrapene carolina carolina* | *Vireo olivaceus* | Bird | Swarth, 2005 |
| *Terrapene carolina carolina* | *Seiurus aurocapillus* | Bird | Swarth, 2005 |
| **Aves** |  |  |  |
| Accipitridae |  |  |  |
| *Accipiter cirrocephalus* | Not id | Bird | Recher, 1985 |
| *Accipiter fasciatus* | Not id | Bird | Recher, 1985 |
| *Accipiter tachiro* | *Phvllastrephus cabanisi* | Bird | Brooks, 2000 |
| *Accipiter tachiro* | *Nectarinia olivacea* | Bird | Brooks, 2000 |
| *Leucopternis albicollis* | *Momotus momota* | Bird | Komar, 2003 |
| *Leucopternis albicollis* | *Catharus ustulatus* | Bird | Komar, 2003 |
| *Leucopternis kuhli* | *Epinecrophylla leucophthalma* | Bird | Anjos et al., 2019 |
| *Rupornis magnirostris* | *Myiothlypis flaveola* | Bird | Ruiz-Esparza, 2012 |
| *Rupornis magnirostris* | *Turdus leucomelas* | Bird | Curcino et al, 2009 |
| Cuculidae |  |  |  |
| *Crotophaga ani* | *Volatinia jacarina* | Bird | Gill & Stokes, 1971 |
| *Geococcyx californianus* | *Cardinalis cardinalis* | Bird | Barclay, 1977 |
| *Geococcyx californianus* | *Junco hyemalis* | Bird | Barclay, 1977 |
| Falconidae |  |  |  |
| *Falco sparverius* | *Riparia riparia* | Bird | Freer, 1985 |
| Falconidae | *Sittasomus griseicapillus* | Bird | Guimarães et al., 2020 |
| Falconidae | *Sciaphylax hemimelaena* | Bird | Guimarães et al., 2020 |
| Falconidae | *Turdus leucomelas* | Bird | Melo et al., 2018 |
| Falconidae | *Vireo chivi* | Bird | Melo et al., 2018 |
| *Micrastur semitorquatus* | *Celeus flavescens* | Bird | Melo et al., 2018 |
| Alcedinidae |  |  |  |
| *Dacelo novaeguineae* | *Eopsaltria australis* | Bird | Recher, 1985;  Armstrong, 2000 |
| *Dacelo novaeguineae* | *Malanus lamberti* | Bird | Recher, 1985;  Armstrong, 2000 |
| *Dacelo novaeguineae* | *Climateris leucophaea* | Bird | Armstrong, 2000 |
| *Dacelo novaeguineae* | *Phylidonyris novaehollandiae* | Bird | Recher, 1985;  Armstrong, 2000 |
| *Dacelo novaeguineae* | *Acanthorhynchus tenuirostri* | Bird | Recher, 1985 |
| *Dacelo novaeguineae* | *Melithreptus lunatus* | Bird | Recher, 1985 |
| Strigidae |  |  |  |
| *Bubo virginianus* | *Myotis lucifugus* | Bat | Jung et al., 2011 |
| *Glaucidium brasilianum* | *Habia fuscicauda* | Bird | Villegas, 2021 |
| *Lophostrix cristata* | *Carollia perspicillata* | Bat | Rocha & Lopez-Baucells, 2014 |
| *Megascops watsonii* | *Carollia brevicauda* | Bat | Serra-Gonçalves et al., 2017 |
| *Pulsatrix perspicillata* | *Nyctinomops laticaudatus* | Bat | Carvalho et al²., 2011 |
| *Pulsatrix perspicillata* | *Phyllostomus hastatus* | Bat | LADIM |
| *Pulsatrix perspicillata* | *Artibeus lituratus* | Bat | LADIM |
| *Pulsatrix perspicillata* | *Carollia perspicillata* | Bat | LADIM |
| *Pulsatrix perspicillata* | *Anoura caudifer* | Bat | LADIM |
| **Mammalia** |  |  |  |
| Didelphimorphia |  |  |  |
| *Chironectes minimus* | *Sturnira lilium* | Bat | Breviglieri & Pedro, 2010 |
| *Chironectes minimus* | *Carollia perspicillata* | Bat | Breviglieri & Pedro, 2010 |
| *Didelphis albiventris* | *Artibeus lituratus* | Bat | Gazarini et al., 2008 |
| *Didelphis albiventris* | *Sturnira lilium* | Bat | Gazarini et al., 2008 |
| *Didelphis aurita* | *Desmodus rotundus* | Bat | LADIM |
| *Didelphis aurita* | *Artibeus obscurus* | Bat | LADIM |
| *Didelphis aurita* | *Artibeus lituratus* | Bat | LADIM |
| *Didelphis marsupialis* | *Artibeus planirostris* | Bat | Castro et al., 2010 |
| *Marmosa demerarae* | *Carollia brevicauda* | Bat | Alencastre-Santos et al., 2022 |
| *Marmosops incanus* | *Artibeus lituratus* | Bat | Faria & Barros, 2014 |
| *Monodelphis* sp. | *Carollia perspicillata* | Bat | LADIM |
| *Philander frenatus* | *Anoura caudifer* | Bat | Patrício-Costa et al., 2008 |
| *Philander frenatus* | *Desmodus rotundus* | Bat | Patrício-Costa et al., 2008 |
| *Philander frenatus* | *Myotis* sp. | Bat | Patrício-Costa et al., 2008 |
| *Philander opossum* | *Artibeus planirostris* | Bat | Castro et al., 2010 |
| Artiodactyla |  |  |  |
| *Odocoileus virginianus* | *Catharus guttatus* | Bird | Allan, 1977 |
| *Odocoileus virginianus* | *Junco hyemalis* | Bird | Allan, 1977 |
| *Odocoileus virginianus* | Not id | Bird | Allan, 1977 |
| Carnivora - Canidae |  |  |  |
| *Cerdocyon thous* | *Pygoderma bilabiatum* | Bat | Novaes et al., 2010 |
| *Cerdocyon thous* | *Carollia perspicillata* | Bat | Novaes et al., 2010 |
| *Cerdocyon thous* | Not id | Bat | Novaes et al., 2010 |
| Carnivora - Felidae |  |  |  |
| *Felis catus* | *Artibeus lituratus* | Bat | Breviglieri & Pedro, 2010 |
| *Felis catus* | *Sturnira lilium* | Bat | Breviglieri & Pedro, 2010 |
| *Felis catus* | *Carollia perspicillata* | Bat | Breviglieri & Pedro, 2010 |
| *Felis catus* | *Glossophaga soricina* | Bat | LADIM |
| *Leopardus wiedii* | *Artibeus fimbriatus* | Bat | Rocha-Mendes & Bianconi, 2009 |
| *Leopardus wiedii* | *Artibeus jamaicensis* | Bat | Gallego et al., 2020 |
| *Leopardus wiedii* | *Artibeus lituratus* | Bat | Rocha-Mendes & Bianconi, 2009 |
| *Leopardus wiedii* | *Sturnira lilium* | Bat | Rocha-Mendes & Bianconi, 2009 |
| *Leopardus wiedii* | Not id | Bat | Rocha-Mendes & Bianconi, 2009 |
| Carnivora - Viverridae |  |  |  |
| *Genetta genetta* | *Miniopterus schreibersii* | Bat | Mas et al., 2015 |
| Chiroptera |  |  |  |
| *Chrotopterus auritus* | *Tadarida brasiliensis* | Bat | Brito et al., 2010 |
| *Chrotopterus auritus* | *Carollia perspicillata* | Bat | Nogueira et al., 2006 |
| *Desmodus rotundus* | *Glossophaga soricina* | Bat | Souza et al., 2011 |
| *Phyllostomus hastatus* | *Desmodus rotundus* | Bat | LADIM |
| *Phyllostomus hastatus* | *Carollia perspicillata* | Bat | Oprea et al., 2006 |
| *Phyllostomus hastatus* | *Myotis nigricans* | Bat | Oprea et al., 2006 |
| *Phyllostomus hastatus* | *Glossophaga soricina* | Bat | Oprea et al., 2006 |
| Primates |  |  |  |
| *Plecturocebus toppini* | *Automolus ochrolaemus* | Bird | Guimarães et al, 2020 |
| *Plecturocebus toppini* | Not id | Bird | Guimarães et al, 2020 |
| *Plecturocebus toppini* | *Onychorhynchus coronatus* | Bird | Guimarães et al, 2020 |
| *Saguinus midas* | *Turdus leucomelas* | Bird | Hilário et al., 2017 |
| *Callithrix jacchus* | *Tolmomyias flaviventris* | Bird | Hilário et al., 2017 |
| *Callithrix jacchus* | *Turdus leucomelas* | Bird | Melo et al., 2018 |
| *Callithrix jacchus* | Not id | Bird | Hilário et al., 2017 |
| *Callitrix penicillata* | *Turdus rufiventris* | Bird | Melo et al., 2018 |
| *Cercopithecus mitis* | *Andropadus latirostris* | Bird | Brooks, 2000 |
| *Cercopithecus mitis* | *Eurillas latirostris* | Bird | Brooks, 2000 |
| *Cercopithecus mitis* | *Andropadus nigriceps* | Bird | Brooks, 2000 |
| *Cercopithecus mitis* | *Brachypterus lopezi* | Bird | Brooks, 2000 |
| *Cercopithecus mitis* | *Trochocercus albonotatus* | Bird | Brooks, 2000 |
| *Cercopithecus mitis* | *Chlorophoneus nigrifrons* | Bird | Brooks, 2000 |
| *Cercopithecus mitis* | *Mandingoa nitidula* | Bird | Brooks, 2000 |
| *Cercopithecus mitis* | Not id | Bird | Brooks, 2000 |
| Not id |  |  |  |
| Not id | *Coryphospingus pileatus* | Bird | Ruiz-Esparza, 2012 |
| Not id | *Cyclarhis gujanensis* | Bird | Ruiz-Esparza, 2012 |
| Not id | *Geotrygon montana* | Bird | Guimarães et al, 2020 |
| Not id | *Hemitriccus margaritaceiventer* | Bird | Ruiz-Esparza, 2012 |
| Not id | *Hylophilus poicilotis* | Bird | Ruiz-Esparza, 2012 |
| Not id | *Isleria hauxwelli* | Bird | Guimarães et al, 2020 |
| Not id | *Leptotila verreauxi* | Bird | Ruiz-Esparza, 2012 |
| Not id | *Myiarchus tyrannulus* | Bird | Ruiz-Esparza, 2012 |
| Not id | *Myiothlypis flaveola* | Bird | Ruiz-Esparza, 2012 |
| Not id | *Nectarinia olivacea* | Bird | Brooks, 2000 |
| Not id | *Pipra fasciicauda* | Bird | Guimarães et al, 2020 |
| Not id | *Pogonocichla stellatai* | Bird | Brooks, 2000 |
| Not id | *Schistochlamys ruficapillus* | Bird | Ruiz-Esparza, 2012 |
| Not id | *Sciaphylax hemimelaena* | Bird | Guimarães et al, 2020 |
| Not id | *Sittasomus griseicapillus* | Bird | Ruiz-Esparza, 2012 |
| Not id | *Thamnophilus pelzelni* | Bird | Ruiz-Esparza, 2012 |
| Not id | *Tolmomyias flaviventris* | Bird | Ruiz-Esparza, 2012 |
| Not id | *Turdus amourochalinus* | Bird | Ruiz-Esparza, 2012 |
| Not id | *Andropadus latirostris* | Bat | Brooks, 2000 |
| Not id | *Artibeus fimbriatus* | Bat | Rocha-Mendes & Bianconi, 2009 |
| Not id | *Artibeus lituratus* | Bat | Rocha-Mendes & Bianconi, 2009 |
| Not id | *Artibeus sp.* | Bat | LADIM |
| Not id | *Carollia perspicillata* | Bat | LADIM |
| Not id | *Molossus molossus* | Bat | LADIM |
| Not id | *Sturnira lilium* | Bat | LADIM |
| Not id | *Trachops cirrhosus* | Bat | LADIM |
